# Association of *ADORA1* rs2228079 and *ADORA2A* rs5751876 Polymorphisms with Gilles de la Tourette Syndrome in the Polish Population

**DOI:** 10.1371/journal.pone.0136754

**Published:** 2015-08-28

**Authors:** Piotr Janik, Mariusz Berdyński, Krzysztof Safranow, Cezary Żekanowski

**Affiliations:** 1 Department of Neurology, Medical University of Warsaw, Warsaw, Poland; 2 Department of Neurodegenerative Disorders, Mossakowski Medical Research Centre, Polish Academy of Sciences, Warsaw, Poland; 3 Department of Biochemistry and Medical Chemistry, Pomeranian Medical University, Szczecin, Poland; Inserm U837, FRANCE

## Abstract

**Background:**

Gilles de la Tourette syndrome (GTS) is a neurodevelopmental disorder characterized by motor and vocal tics. Hyperactivity of dopaminergic transmission is considered a prime abnormality in the pathophysiology of tics. There are reciprocal antagonistic interactions between adenosine and dopamine transmission. The aim of the study was to analyze the association of two polymorphisms, rs2228079 in *ADORA1* and rs5751876 in *ADORA2A*, with the risk of GTS and co-morbid disorders.

**Material and Methods:**

A total of 162 Polish GTS patients and 270 healthy persons were enrolled in the study. Two polymorphisms were selected on the basis of knowledge of SNPs frequencies in *ADORA1* and *ADORA2A*. Chi-square test was used for allelic and genotypic association studies. Association of genotypes with age of tic onset was analyzed with Mann-Whitney test. Multivariate logistic regression was used to find independent predictors of GTS risk.

**Results:**

We found that the risk of GTS was associated with rs2228079 and rs5751876 polymorphisms. The GG+GT genotypes of rs2228079 in *ADORA1* were underrepresented in GTS patients (p = 0.011), whereas T allele of rs5751876 in *ADORA2A* was overrepresented (p = 0.017). The GG genotype of rs2228079 was associated with earlier age of tic onset (p = 0.046). We found also that the minor allele G of rs2228079 was more frequent in GTS patients with depression as compared to the patients without depression (p = 0.015). Also the genotype GG was significantly more frequent in patients with obsessive compulsive disorder/behavior (OCD/OCB, p = 0.021) and depression (p = 0.032), as compared to the patients without these co-morbidities. The minor allele T frequency of rs5751876 was lower in GTS patients with co-morbid attention deficit hyperactivity disorder (p = 0.022), and TT+TC genotypes were less frequent in the non-OCD anxiety disorder group (p = 0.045).

**Conclusion:**

*ADORA1* and *ADORA2A* variants are associated with the risk of GTS, co-morbid disorders, and may affect the age of tic onset.

## Introduction

Gilles de la Tourette syndrome (GTS) is a neuropsychiatric disorder manifesting by multiple motor tics and one or more vocal tics, lasting longer than a year, with the age of onset <18 years [1]. In addition to tics, many patients with GTS exhibit a variety of mental disorders and behavioral symptoms such as attention deficit hyperactivity disorder (ADHD), obsessive compulsive disorder (OCD), poor impulse control, conduct disorder, oppositional defiant disorder, anxiety, depression, temper outbursts, rage attacks, self-injuries, and inappropriate sexual behavior [2, 3]. Although the etiology of GTS remains unknown, a predominant contribution of genetic factor is acknowledged [4–7]. Susceptibility genes associated with GTS include: Slit and Trk-like 1 (*SLITRK1*), L-histidine decarboxylase (*HDC*), mitochondrial inner membrane protease subunit 2 (*IMMP2L*), neuroligin 4, X-linked (*NLGN4X)*, and contactin-associated protein 2 (*CNTNAP2*) [8–12]. However, these genes contribute to a minority of GTS cases only and a substantial role of environmental factors such as infections, neuroimmunological disturbances, prenatal and peri-natal complications, psychosocial stressors and androgen influences have also been established [13–17].

The pathophysiology of tics and co-morbid mental disorders remains poorly understood. Evidence supports an involvement of the cortico-striatal-thalamo-cortical (CSTC) circuit in GTS pathology, however, the primary anatomical site of the abnormality remains controversial. The dysfunction of neurotransmission within the CSTC circuit is known to play a significant role in the pathophysiology of tics. Although numerous neurotransmitters participate in CSTC signal transmission, dopaminergic dysfunction is considered the prime abnormality in GTS since dopamine antagonists bring about tic suppression. Still, several lines of evidence support a role of other neurotransmitters in the pathogenesis of tics and associated psychiatric disorders [18–21].

Adenosine is an important modulator of striatal neurotransmission whose role in GTS has not been thoroughly investigated yet. In the nervous system adenosine acts as an energy dependent neuromodulator through combined presynaptic, postsynaptic and non-synaptic actions [22, 23]. Extracellular adenosine regulates several functions in the brain, including neuronal viability, membrane potential, propagation of action potentials, and astrocytic function [22]. Adenosine acts through four subtypes of receptors: A1, A2A, A2B, and A3. A1 and A2A receptors are predominantly expressed in the brain and are largely responsible for the central effects of adenosine [24, 25]. The most abundant and homogeneously distributed adenosine receptor in the brain is A1, whose physiological action involves inhibition of synaptic transmission and neuron hyperpolarization [26]. In contrast, the A2A receptor is expressed at a high level only in the striatum, the olfactory tubercle and the nucleus accumbens and its functions in the brain include facilitation of neurotransmitter release and regulation of sensorimotor integration in basal ganglia [27]. Thus, these two adenosine receptors have opposite physiological actions. Interestingly, they have been shown to form heterodimers where A2A receptor can control the A1 receptor functionality, whereas the reverse does not occur [28].

A1 and A2 receptors have been found in dopamine-rich regions of the brain and co-localized with dopamine D1 and D2 receptors on striatopallidal GABAergic neurons. This co-localization of adenosine and dopamine receptors allows their direct interactions in the membrane through formation of heteromeric complexes and also a cross-talk in intracellular signaling [29–31]. A1-D1 receptor interaction would modulate the functioning of the medium spiny striatal neurons of the direct pathway, whereas A2-D2 receptor interaction would affect transmission through the indirect pathway of the striatum [32, 33]. Additionally, most evidence suggests that A1 and D1, and A2A and D2 receptors act antagonistically to regulate GABA-ergic striatal output neurons. Stimulation of A1 and A2A receptors counteracts, respectively, D1- and D2- receptor-mediated neurotransmission, tending to reduce locomotor activity, and antagonism of either of these two adenosine receptors promotes locomotor activity [34–36]. The antagonistic interactions between the two receptors have also been shown at the level of adenylyl cyclase, with D2 receptor preventing A2A receptor-mediated protein phosphorylation and gene expression [28, 31, 37]. This antagonism has implications for the treatment of Parkinson’s disease which is associated with reduced level of dopamine in the striatum. A2A receptor antagonists have the potential to be effective for the treatment of Parkinson’s disease [38, 39]. In contrast to Parkinson’s disease, GTS is considered to be related to a hyperdopaminergic state within the CSTC circuit.

In the present study we tested the hypothesis that the increased dopaminergic neurotransmission in GTS could be related to variants of *ADORA1* and *ADORA2A* genes. We analyzed the association of *ADORA1* rs2228079 and *ADORA2A* rs5751876 polymorphisms (selected as described below) with the incidence and clinical phenotype of GTS.

## Material and Methods

### Ethics Statement

The study has been approved by the Ethics Committee of Medical University of Warsaw (KB/2/2007, KB/53/A/2010), and has therefore been performed in accordance with the ethical standards laid down in the 1964 Declaration of Helsinki and its later amendments. All participants aged ≥18 years signed informed consent form prior to their inclusion in the study, and their legal guardians gave a written consent on behalf of individuals under 18.

### Study participants

The cohort of GTS cases comprised 162 unrelated patients aged 4–54 years (mean age: 19.9 ± 8.7 years; 131 males (80.9%), 75 children and 87 adults defined as ≥18 years, 53.7%). The family history was positive in 80 (49.7%) patients, and was unknown for one patient. The mean age of tic onset was 7.5±3.2 years. One-hundred and twenty five (77.2%) patients had at least one of the following co-morbidities: ADHD n = 63 (38.9%), OCD/OCB n = 70 (43.2%), learning disorder n = 51 (31.5%), depression n = 25 (15.4%), non-OCD anxiety disorder n = 34 (21.0%), conduct disorder n = 12 (7.4%), while 37 (22.8%) had none of the above. As a control group we enrolled 270 unrelated, ethnically and gender matched individuals with no diagnosed mental, neurological or general disorder, aged 12–53 years (mean age: 21.5 ± 3.0 years; 200 males, 74.1%). The patients were evaluated for the clinical diagnosis of GTS and co-morbid psychiatric disorders at the time of examination according to DSM-IV-TR [1]. Learning disorders were diagnosed if they had been confirmed by psychologist’s evaluation. Obsessive Compulsive Behavior (OCB) was diagnosed if obsessions and compulsions were egosyntonic in contrast to egodystonic symptoms which characterized OCD. The diagnosis of co-morbid mental disorders was also made based on earlier psychiatric examinations that had been performed before the time of patients’ evaluation. However, for some adults the earlier medical records and the results of psychiatric examinations were not available. In those patients some data were retrospective based on reports from patients.

### Genetic analysis and selection of SNPs

Genomic DNA was extracted from peripheral blood leukocytes using standard salting-out method. Intronic primers were used to amplify all coding exons of *ADORA1* (2 and 3 according to ENST00000367236) and all coding exons of *ADORA2A* (2 and 3 according to ENST00000337539) in a subgroup of 50 GTS patients (Table in S1 Table). Amplicons were purified with Exonuclease I / FastAP (Fermentas) and sequenced on an ABI PRISM 3130 Genetic Analyzer (Applied Biosystems) using the BigDye Terminator v1.1 Cycle Sequencing Kit (Applied Biosystems).

Sequence analysis of coding exons and exon-intron boundaries in the 50 GTS patients revealed only two single synonymous polymorphisms: one in *ADORA1* exon 2 (rs2228079, c.306T>G, A102A) and one in *ADORA2A* exon 3 (rs5751876, c.1083T>C, Y361Y). *ADORA1 rs2228079* and *ADORA2A rs5751876* polymorphisms were chosen on the basis of their relative frequencies in the general population, as well as in a subgroup of 50 Polish GTS patients. More specifically, we analyzed coding sequences of exons 2 and 3 (both in the *ADORA1* and *ADORA2A* genes). We identified only two SNPs, i.e. *ADORA1* rs2228079 and *ADORA2A* rs5751876. It is consistent with our unpublished results of analysis of the whole exome of one hundred Polish subjects not having GTS which revealed only the aforementioned two SNPs in *ADORA1* and *ADORA2A* genes. Additionally, data presented by Exome Aggregation Consortium (ExAC) suggest that other exonic variants of the *ADORA1* and *ADORA2A* genes are private ones with a frequency up to dozens per 100,000 alleles. Thus the performed analyses prompted us to confine the association study to the two silent exonic SNPs in *ADORA1* and *ADORA2A* (rs2228079 and rs5751876, respectively). As in the case of other genetic association studies it is possible that both silent variants are in linkage disequilibrium with true risk conferring variants located in the not analyzed intronic sequences. It is also possible that the SNPs could influence putative epigenetic mechanisms. Both polymorphisms were then determined in the whole group of patients and controls, using the same fluorescent sequencing method, however, confined to SNP-bearing exons (i.e. *ADORA1* exon 2 and *ADORA2A* exon 3).

### Statistical analysis

Chi-square test was used for both allelic and genotypic association studies. Association of genotypes with age of tic onset was analyzed with Mann-Whitney test. Multivariate logistic regression model was used to find independent predictors of GTS risk. The study sample size was sufficient to detect with 80% probability the true effect size measured as odds ratio (OR) equal to 1.50 or 0.65 for the differences in allele frequencies of the two polymorphisms between GTS and control group. Statistica 10 program was used for statistical calculations. The significance level was set at p < 0.05.

## Results

The genotype frequencies of both SNPs were in accordance with the Hardy–Weinberg equilibrium both in the control and patient groups (p>0.1 for rs2228079, p>0.3 for rs5751876).

### Incidence of GTS

Significant associations were found between variants of the *ADORA1* and *ADORA2A* genes and the incidence of GTS. The minor allele frequency (MAF) of *ADORA1* rs2228079 was lower but not significantly (p = 0.069), and of *ADORA2A* rs5751876 was significantly higher in GTS patients compared to control group (Table 1). When the TT genotype was used as a reference, *ADORA1* rs2228079 genotypes GG+GT (dominant model, p = 0.011) and GT (p = 0.008) were statistically significantly associated with lower risk of GTS. The presence of *ADORA2A* rs5751876 genotype TT (recessive model) and TT+TC (dominant model) was associated with higher risk of GTS (Table 1). Multivariate logistic regression analysis adjusted for gender showed that the presence of *ADORA1* rs2228079 allele G (genotypes GG+GT) is an independent factor associated with lower risk of GTS, while a higher number of *ADORA2A* rs5751876 T alleles is associated with a higher risk of GTS (Table 2).

**Table 1:** Genotype and allele distribution of the rs2228079 and the rs5751876 in patients with Gilles de la Tourette syndrome and controls.

*ADORA1* rs2228079											
Group		Genotype			Allele						
		TT	GT	GG	T	G	Comparison ^a^	OR ^b^	95%CI		p ^c^
Controls	n	87	144	39	318	222	GG+GT vs TT	0.594	0.398	0.888	0.011
	%	32.2	53.3	14.4	58.9	41.1	GG vs GT+TT	0.980	0.562	1.710	0.940
GTS	n	72	67	23	211	113	G vs T	0.767	0.577	1.021	0.069
	%	44.4	41.4	14.2	65.1	34.9					
*ADORA2A* rs5751876											
Group		Genotype			Allele						
		CC	TC	TT	C	T	Comparison ^a^	OR ^b^	95%CI		p ^c^
Controls	n	114	129	27	357	183	TT+TC vs CC	1.503	0.999	2.259	0.049
	%	42.2	47.8	10.0	66.1	33.9	TT vs TC+CC	1.800	1.015	3.194	0.043
GTS	n	53	82	27	188	136	T vs C	1.411	1.063	1.874	0.017
	%	32.7	50.6	16.7	58.0	42.0					

^a^ Comparison of genotype or allele frequencies between the GTS and control groups

^b^ OR for the genotype or allele frequencies compared between GTS patients and controls

^c^ Chi^2^ test

**Table 2:** Multivariate logistic regression analysis of independent factors associated with presence of GTS as dependent variable (GTS patients vs controls)

Independent variables	OR	95% CI		p
Gender (male vs female)	1.394	0.855	2.273	0.180
*ADORA1* rs2228079 (GG+GT vs TT)	0.571	0.380	0.859	0.007
*ADORA2A* rs5751876 (number of T alleles) ^a^	1.436	1.062	1.941	0.018

^a^ OR value for each additional T allele

### Co-morbidity

Allelic and genotypic association of both analyzed SNPs with co-morbid disorders were shown in Table 3. The minor allele G of rs2228079 was more frequent in GTS patients with depression than in patients without depression (OR 2.11, 95% CI:1.15–3.89, p = 0.015). The MAF of *ADORA2A* rs5751876 (allele T) was lower in GTS patients with ADHD compared to those without ADHD (OR 0.59, 95% CI 0.37–0.93, p = 0.022). The genotype GG of *ADORA1* rs2228079 (recessive model) was significantly more frequent in patients with OCD/OCB (OR 2.86, 95% CI: 1.14–7.21, p = 0.021) and depression (OR 2.94, 95% CI: 1.06–8.13, p = 0.032) than in patients without these co-morbidities. There was also a non-significant trend toward an association between the GG genotype in *ADORA1* rs2228079 and non-OCD anxiety disorders. *ADORA2A* rs5751876 genotypes TT+TC (dominant model) were significantly associated with a lower risk of non-OCD anxiety disorders (OR 0.46, 95% CI: 0.21–1.0, p = 0.045). All mentioned significant allelic and genotypic associations with the GTS co-morbidities remained statistically significant in multivariate logistic regression analysis adjusted for gender (data not shown).

**Table 3:** Genotypic and allelic association analysis of *ADORA1* rs2228079 and *ADORA2A* rs5751876 variants with co-morbid disorders in patients with Gilles de la Tourette syndrome.

*ADORA1* rs2228079											
Co-morbid disorder		Genotype			Allele						
(- absent, + present)		TT	GT	GG	T	G	Comparison ^a^	OR ^b^	95%CI		p ^c^
No co-morbidity	n	17	16	4	50	24	GG+GT vs TT	1.082	0.518	2.260	0.834
	%	45.9	43.2	10.8	67.6	32.4	GG vs GT+TT	1.479	0.470	4.655	0.502
Any co-morbidity	n	55	51	19	161	89	G vs T	1.152	0.664	1.999	0.616
	%	44.0	40.8	15.2	64.4	35.6					
ADHD -	n	42	40	17	124	74	GG+GT vs TT	0.811	0.429	1.530	0.517
	%	42.4	40.4	17.2	62.6	37.4	GG vs GT+TT	0.508	0.189	1.367	0.174
ADHD +	n	30	27	6	87	39	G vs T	0.751	0.467	1.208	0.237
	%	47.6	42.9	9.5	69.1	31.0					
OCD/OCB -	n	42	42	8	126	58	GG+GT vs TT	1.120	0.599	2.095	0.723
	%	45.7	45.7	8.7	68.5	31.5	GG vs GT+TT	2.864	1.138	7.207	0.021
OCD/OCB +	n	30	25	15	85	55	G vs T	1.406	0.887	2.227	0.146
	%	42.9	35.7	21.4	60.7	39.3					
Learning Disorder-	n	46	48	17	140	82	GG+GT vs TT	0.680	0.349	1.325	0.256
	%	41.4	43.2	15.3	63.1	36.9	GG vs GT+TT	0.737	0.272	1.996	0.548
Learning Disorder +	n	26	19	6	71	31	G vs T	0.745	0.451	1.232	0.251
	%	51.0	37.3	11.8	69.6	30.4					
Depression-	n	65	56	16	186	88	GG+GT vs TT	2.321	0.911	5.915	0.072
	%	47.5	40.9	11.7	67.9	32.1	GG vs GT+TT	2.941	1.064	8.130	0.032
Depression +	n	7	11	7	25	25	G vs T	2.114	1.149	3.888	0.015
	%	28.0	44.0	28.0	50.0	50.0					
Non-OCD Anxiety Disorder -	n	54	59	15	167	89	GG+GT vs TT	0.649	0.304	1.386	0.262
	%	42.2	46.1	11.7	65.2	34.8	GG vs GT+TT	2.318	0.889	6.043	0.079
Non-OCD Anxiety Disorder +	n	18	8	8	44	24	G vs T	1.023	0.585	1.792	0.935
	%	52.9	23.5	23.5	64.7	35.3					
Conduct Disorder -	n	64	65	21	193	107	GG+GT vs TT	0.372	0.107	1.290	0.107
	%	42.7	43.3	14.0	64.3	35.7	GG vs GT+TT	1.229	0.251	6.005	0.799
Conduct Disorder +	n	8	2	2	18	6	G vs T	0.601	0.232	1.560	0.291
	%	66.7	16.7	16.7	75.0	25.0					
*ADORA2A* rs5751876											
Co-morbid disorder		Genotype			Allele						
(- absent, + present)		CC	TC	TT	C	T	Comparison ^a^	OR ^b^	95%CI		p ^c^
No co-morbidity	n	9	22	6	40	34	TT+CT vs CC	0.592	0.256	1.365	0.216
	%	24.3	59.5	16.2	54.1	46.0	TT vs CT+CC	1.043	0.387	2.813	0.933
Any co-morbidity	n	44	60	21	148	102	T vs C	0.811	0.481	1.367	0.431
	%	35.2	48.0	16.8	59.2	40.8					
ADHD -	n	26	53	20	105	93	TT+CT vs CC	0.475	0.243	0.928	0.028
	%	26.3	53.5	20.2	53.0	47.0	TT vs CT+CC	0.494	0.196	1.247	0.130
ADHD +	n	27	29	7	83	43	T vs C	0.585	0.368	0.928	0.022
	%	42.9	46.0	11.1	65.9	34.1					
OCD/OCB -	n	30	46	16	106	78	TT+CT vs CC	0.989	0.510	1.918	0.973
	%	32.6	50.0	17.4	57.6	42.4	TT vs CT+CC	0.886	0.382	2.051	0.777
OCD/OCB +	n	23	36	11	82	58	T vs C	0.961	0.616	1.501	0.862
	%	32.9	51.4	15.7	58.6	41.4					
Learning Disorder-	n	33	61	17	127	95	TT+CT vs CC	0.656	0.328	1.313	0.232
	%	29.7	55.0	15.3	57.2	42.8	TT vs CT+CC	1.349	0.569	3.196	0.496
Learning Disorder +	n	20	21	10	61	41	T vs C	0.899	0.558	1.447	0.660
	%	39.2	41.2	19.6	59.8	40.2					
Depression-	n	43	70	24	156	118	TT+CT vs CC	0.686	0.285	1.651	0.399
	%	31.4	51.1	17.5	56.9	43.1	TT vs CT+CC	0.642	0.178	2.319	0.496
Depression +	n	10	12	3	32	18	T vs C	0.744	0.398	1.389	0.352
	%	40.0	48.0	12.0	64.0	36.0					
Non-OCD Anxiety Disorder -	n	37	70	21	144	112	TT+CT vs CC	0.457	0.211	0.992	0.045
	%	28.9	54.7	16.4	56.3	43.8	TT vs CT+CC	1.092	0.402	2.962	0.863
Non-OCD Anxiety Disorder +	n	16	12	6	44	24	T vs C	0.701	0.402	1.222	0.209
	%	47.1	35.3	17.7	64.7	35.3					
Conduct Disorder -	n	50	74	26	174	126	TT+CT vs CC	1.500	0.389	5.786	0.554
	%	33.3	49.3	17.3	58.0	42.0	TT vs CT+CC	0.434	0.054	3.506	0.421
Conduct Disorder +	n	3	8	1	14	10	T vs C	0.986	0.424	2.292	0.975
	%	25.0	66.7	8.3	58.3	41.7					

^a^ Comparison of genotype or allele frequencies between GTS patients with and without particular co-morbid disorder

^b^ OR for the genotype or allele frequencies compared between GTS patients with and without particular co-morbid disorder

^c^ Chi^2^ test

### Gender

A higher frequency of *ADORA2A* rs5751876 T allele was found in males than in females, and this difference was statistically significant in controls (36.5% vs 26.4%, p = 0.030) but not in GTS patients (44.3% vs 32.3%, p = 0.085). No association between gender and *ADORA1* genotype was found.

### Age of tic onset

The GG genotype of *ADORA1* rs2228079 was significantly associated with an earlier age at onset of the disease—GG vs. GT+TT: 6.3±3.0 vs 7.7±3.2 years, p = 0.046. This association remained significant (p = 0.025) in multivariate linear regression analysis adjusted for gender.

## Raw data

The raw data underlying the findings of the study are presented in Table in S2 Table.

## Discussion

The dysfunction of neurotransmission in the cortex and the basal ganglia, mainly overactivity of the dopaminergic system, plays a significant role in the pathophysiology of tics. According to a classical basal ganglia model, hyperkinetic disorders such as tics are a result of an increased cortical excitability due to either a reduction of the indirect pathway transmission or an increase of the direct pathway signalling [18–21]. Enkephalinergic neurons of the indirect pathway express predominantly A2A-D2 heteromeric receptor complexes, while dynorphinergic neurons of the direct pathway express predominantly A1-D1 heteromers. There is a mutual antagonistic interaction between the two receptors forming a heterodimer, thus stimulation of one receptor decreases the binding capacity of the other [32–36]. Another fact that has drawn our attention to abnormal functioning of adenosine receptors as a possible contribution to the pathophysiology of tics is the clinical observation that consumption of caffeine-rich products such as coffee, certain soft drinks (e.g. coke) and black tea may deteriorate tics in some GTS patients [40]. Caffeine is a competitive antagonist of adenosine receptors and enhances dopaminergic activity [41]. Therefore we decided to analyze the contribution of *ADORA1* and *ADORA2A* variants in a group of Polish GTS patients and controls.

A significant association was found both in the allelic and genotypic analysis between the risk of GTS and the examined SNPs of the genes coding for adenosine A1 and A2 receptors (Table 1). Multivariate analysis proved that both SNPs are gender-independent factors associated with a lower (*ADORA1* rs2228079 GG+GT genotypes, dominant model) or higher (*ADORA2A* rs5751876 T allele, additive model) GTS risk (Table 2). We also found that a variant of *ADORA1* could be related to an earlier age of tic onset. We hypothesize that the binding affinity of A1 and A2A receptors for adenosine could depend on the variants of the encoding genes. Although both variants examined are synonymous they could affect gene expression. The most likely explanation is a linkage to functional variants located in coding and/or regulatory regions. It has also been shown that synonymous variants do influence mRNA stability and translation rate [42]. For instance, the rs5751876 polymorphism in the Japanese population is completely linked with three other variants (rs2298383, rs5751876, and rs35320474), which results in two haplotypes (A and B). Haplotype AA (with allele T of rs5751876) has a higher risk of developing acute encephalopathy with biphasic seizures and late reduced diffusion (AESD). Moreover, mRNA expression in the brain is significantly higher in AA than in AB and BB individuals of Caucasian background. Similarly, ADORA2A protein level and cellular cAMP production are significantly higher in the AA than in the BB haplotype carriers. The rs5751876 polymorphism is also linked with variants likely to alter the level of mRNA i.e. rs22983836 located in the putative promoter region with regulatory element predicted *in silico* and rs35320474 located in the 3`UTR including U-rich motifs [43].

Another mechanism affecting ADORA1 protein level based on codon usage bias is also plausible [44]. The GCG codon corresponding to the minor G allele is 2.5-fold less frequently used than the GCT one corresponding to the major allele in humans [45]. This difference is even more pronounced for the *ADORA1* gene (NM_000674.2, GCT frequency 0.34 vs. GCG, 0.09). It is well known that the use of synonymous codons is neither random nor neutral. For instance, synonymous mutations in *DRD2* and *MDR1* genes have been shown to affect mRNA stability and translation [46, 47]. Also *in silico* analysis suggests that codon bias plays an important role in translation efficiency in humans, with significant differences between tissues and developmental stages [48, 49]. Thus it seems likely that translation rate and/or folding of *ADORA1* protein could be modulated via the codon bias mechanism i.e., depend on the rs2228079 polymorphism.

Notably, both variants seem to act in opposite directions. The minor allele G of *ADORA1* rs2228079 could enhance adenosine transmission through A1 receptor and therefore decrease dopamine action *via* the striatal direct pathway, thereby reducing tic severity. In contrast, the minor allele T of *ADORA2A* rs5751876 variant would decrease the affinity of A2A receptor for adenosine, consequently enhancing dopaminergic transmission and thus increasing the risk of GTS. We hypothesize that the functional imbalance between the direct and indirect pathways dependent on the activity of adenosine receptors due to abnormal expression of *ADORA1* and *ADORA2A* genes could be responsible for the appearance of tics. Indeed, multivariate logistic regression analysis (Table 2) suggests that the diplotype comprising *ADORA1* TT and *ADORA2A* TT genotypes is associated with the highest risk of GTS. The proportion of subjects with the TT/TT diplotype is 8.6% in the GTS group and 1.1% in the control group (OR = 8.419, 95%CI = 2.381–29.771, p = 0.00015). This effect could be due to a direct or a functional interaction between A1 and A2A receptors, or both. However, the association should be treated with caution, since the number of TT/TT diplotype individuals is rather low in our study groups.

Several lines of evidence support a potential role of the glutamatergic system in GTS. Extensive interaction between the glutamate and dopamine neurotransmitter systems has been established [21]. A1-A2 receptor heterodimers are located in presynaptic striatal glutamatergic synapses and control the striatal glutamatergic neurotransmission [50–52]. It is possible that abnormal functioning of A1 and A2 receptors may affect the glutamatergic transmission in GTS. Moreover, the existence of oligomeric complexes comprised of adenosine, dopamine and glutamate receptors (e.g. A2A/D2/mGlu5 receptor complex) has also been demonstrated. These receptor oligomers are located on enkephalin medium spiny neurons in the striatum and control their excitability [53, 54]. The A2A/mGlu5 receptor synergism in the rat striatum has been established, and it has been suggested that A2A/mGlu5 receptor co-stimulation blocks D2-receptor-mediated transmission [28]. Thus, down-regulation of the A2A/mGlu5 receptor complex could potentiate D2 receptor activity.

We also found that both polymorphisms studied were associated with co-morbid disorders in GTS patients. The GG genotype of *ADORA1* rs2228079 was found significantly more frequently in patients with OCD/OCB, depression and, at borderline significance, with non-OCD anxiety disorder. OCD is integral to GTS in contrast to depression and non-OCD anxiety disorders, usually considered not to be genetically linked to GTS [55]. Among these factors, OCD has been shown to have a high rate of co-morbidity with depression and non-OCD anxiety disorders [55–58]. The three disorders are common in patients with GTS and the present study has shown that they could have the same genetic background.

It is generally accepted that GTS and ADHD are not genetically related. However, O'Rourke et al. have suggested that the two disorders combined with OCD may in fact have overlapping neurobiology [59]. Indeed, our results agree with the latter hypothesis. We found an association between rs5751876 variant and co-morbid ADHD, a higher number of T alleles of rs5751876 predicting a lower risk of co-occurring ADHD. Interestingly, there was also a similar relationship between *ADORA2A* rs5751876 and non-OCD anxiety disorders, but only in the dominant model: the T allele was associated with a lower risk (Table 3). Thus, we found that non-OCD anxiety disorders were related to variants of the two studied genes: rs2228079 of *ADORA1* (not reaching significance) and rs5751876 of *ADORA2A* (significantly), which is not in line with earlier reports considering anxiety symptoms as secondary to GTS [60].

## Conclusions

We have found a significant association of the SNPs studied with the risk of GTS, co-morbidity, and age of tic onset.

## Limitations

The major limitation of the study is a relatively small sample size and the fact that only protein-coding parts of *ADORA1* and *ADORA2A* genes were analyzed. Thus, the present findings should be verified for larger groups of patients and controls, preferably also for different populations. Moreover, a role of other functional polymorphisms in *ADORA1* and *ADORA2A* cannot be excluded in conferring liability to GTS. The patients were evaluated for co-morbid disorders only once (one-time registration) and we cannot exclude that some new psychiatric disorders might develop over time, especially in young children. The associations of co-morbid conditions with variants of the two studied genes must be interpreted with cautious because of small number of patients with particular psychiatric disorder. The data regarding the age of tic onset and the diagnosis of co-morbid disorders in some adult patients were retrospective, based on reports from parents or patients and were, therefore, subject to recall bias.

## Supporting Information

S1 Table Primers used for PCR amplification and sequencing. (DOCX)

S2 Table Dataset of raw values underlying the findings of the study. (XLS)
