# Supplementary material for: Association of ADORA1 rs2228079 and ADORA2A rs5751876 Polymorphisms with Gilles de la Tourette Syndrome in the Polish Population
Source: PLoS One. 2015 Aug 28;10(8):e0136754. doi: 10.1371/journal.pone.0136754 (PMC4552818; doi:10.1371/journal.pone.0136754)
Supplement: S1 Table — (DOCX) [file pone.0136754.s001.docx]

S1 Table. Primers used for PCR amplification and sequencing.

| *ADORA1* | | |
| --- | --- | --- |
| Exon 2 | Ado1-1f | CCTGACCACACAGGTGCTT |
|  | Ado1-1r | CTCAAGCCAGCCATCATGT |
| Exon 3 | Ado1-2f | CTGGAAGAGGAGGGTGCTC |
|  | Ado1-2r | GCATGTGAGGACTGGACTGA |
| *ADORA2A* | | |
| Exon 2 | Ado2a-1f | ACTTGGCTCCTGTGAGGAAG |
|  | Ado2a-1r | AGCTCACCATGCTGACACTG |
| Exon 3 | Ado2a-2f | CTGGGGTTCTGAACTCTTGG |
|  | Ado2a-2r | TGCTCTGTGGAGACAAGGTG |
|  | Ado2a-2rw* | CTAAGGAGCTCCACGTCTGG |

*Primer used to Sanger sequencing
